# Effects of Spaghetti Differing in Soluble Fiber and Protein Content on Glycemic Responses in Humans: A Randomized Clinical Trial in Healthy Subjects

**DOI:** 10.3390/ijerph19053001

**Published:** 2022-03-04

**Authors:** Emilia Papakonstantinou, Marina Xaidara, Vassiliki Siopi, Marianna Giannoglou, George Katsaros, Georgios Theodorou, Eirini Maratou, Kalliopi-Anna Poulia, George D. Dimitriadis, Panagiotis N. Skandamis

**Affiliations:** 1 Laboratory of Dietetics and Quality of Life, Department of Food Science and Human Nutrition, School of Food and Nutritional Sciences, Agricultural University of Athens, 11855 Athens, Greece; stud516028@aua.gr (M.X.); vickysiopi888@gmail.com (V.S.); lpoulia@gmail.com (K.-A.P.); 2 Institute of Technology of Agricultural Products, Hellenic Agricultural Organization “DEMETER”, 14123 Athens, Greece; giannoglou@chemeng.ntua.gr (M.G.); gkats@chemeng.ntua.gr (G.K.); 3 Laboratory of Animal Breeding and Husbandry, Department of Animal Science, School of Animal Biosciences, Agricultural University of Athens, 11855 Athens, Greece; gtheod@aua.gr; 4 Department of Clinical Biochemistry, “Attikon” University General Hospital, National and Kapodistrian University of Athens, Haidari, 12462 Athens, Greece; maratou@hotmail.com; 5 Sector of Medicine, Medical School, National and Kapodistrian University of Athens, 15772 Athens, Greece; gdimitr@med.uoa.gr; 6 Laboratory of Food Quality Control and Hygiene, Department of Food Science and Human Nutrition, School of Food and Nutritional Sciences, Agricultural University of Athens, 11855 Athens, Greece; pskan@aua.gr

**Keywords:** spaghetti, glycemic index, glycemic response, healthy, soluble fiber, protein, low carbohydrate, pasta

## Abstract

This randomized, single blind, cross-over study investigated the glycemic responses to three spaghetti No 7 types differing in dietary protein and soluble fiber content. Fourteen clinically and metabolically healthy, fasting individuals (25 ± 1 years; ten women; BMI 23 ± 1 kg/m^2^) received isoglucidic test meals (50 g available carbohydrate) and 50 g glucose reference, in random order. GI was calculated using the FAO/WHO method. Capillary blood glucose and salivary insulin samples were collected at 0, 15, 30, 45, 60, and 120 min. Subjective appetite ratings (hunger, fullness, and desire to eat) were assessed by visual analogue scales (VAS, 100 mm) at baseline and 120 min. All three spaghetti types (regular, whole wheat, and high soluble fiber–low carbohydrates) provided low GI values (33, 38, and 41, respectively, on glucose scale) and lower peak glucose values compared to glucose or white bread. No differences were observed between spaghetti No 7 types for fasting glucose, fasting and post-test-meal insulin concentrations, blood pressure (systolic and diastolic), and subjective appetite. Conclusions: all spaghetti No 7 types, regardless of soluble fiber and/or protein content, attenuated postprandial glycemic response, which may offer advantages to glycemic control.

## 1. Introduction

It is well accepted that cereals and their products, particularly pasta, are the principal components of our diet. When starchy foods are consumed blood glucose rises; however, the extent of this rise (increment) depends on the amount of total carbohydrates consumed, the type of carbohydrate, and other components, such as soluble fiber, protein, and fat content of these foods, preparation method, cooking practices, etc. The glycemic index (GI) is a tool developed to systematically classify carbohydrate-containing foods according to time-integrated effects on postprandial glucose response [1,2]. GI is defined as the incremental area under the blood glucose curve (iAUC) elicited by a 50 g available carbohydrate portion of a food expressed as a percentage of that after 50 g carbohydrate from a reference food (typically D-glucose or white bread) taken by the same subject [3,4,5]. The starch of high GI foods is digested and absorbed rapidly from the human digestive system, which may lead to glucose spikes and troughs; whereas carbohydrates in foods with a low GI are slowly digested and absorbed, and as a result, diets with a low GI may be more beneficial in controlling postprandial plasma glucose excursions [6]. Increased glucose fluctuations have been shown to induce oxidative stress and β-cell damage [7]. Additionally, increased glucose variability from peaks to nadirs has been recognized as a major metabolic defect leading to cardiovascular diseases [8]. Consequently, foods containing rapidly digested, absorbed, and metabolized carbohydrates are considered high GI foods, whereas those containing slowly digested, absorbed, and metabolized carbohydrates are considered low GI foods (low GI: ≤55, moderate GI: 56–69, high GI: ≥70 on the glucose scale) [3]. The glycemic load (GL) is a mathematical equation considering the product of GI and the total available carbohydrate content in a food amount [3]. The GL of a food can be classified as low, medium, or high (low: ≤10, medium: 11–19, high: ≥20). It has been proposed that the GL is a good predictor of postprandial glycemia associated with consuming a serving portion of a particular food [9]. It has been shown that regular consumption of high GI foods is associated with increased chronic disease risk [3,10,11], whereas low to moderate GI foods are considered favorable to health [2,12]. Replacement of higher GI with lower GI foods seems to offer a moderate improvement in glycemic control [13,14]. In some cohort studies, the GL, but not the carbohydrate content, has been frequently linked to a reduced risk of type 2 diabetes [10]. It has been proposed that lowering the GL of consumed carbohydrates may lead to a significant reduction in hemoglobin A1C from −0.2% up to −0.5% [14,15,16]. It should be noted however that other factors, such as the inclusion of soluble dietary fiber, resistant starch and amylose, and inclusion of non-cereal ingredients (i.e., οat fiber, flaxseed, legume-based flours, protein) may influence the glycemic responses [3,6,17,18,19,20]. We have shown in a series of studies that foods (such as Ceratonia siliqua, carob) and characteristics (such as large bran size in breads or the sucrose to oligosaccharide ratio in honey varieties of similar botanical origin and characterization explaining 30% of the postprandial glucose differences) are able to reduce glucose excursions and overall postprandial glycemic responses [21,22,23].

Spaghetti is a commonly consumed food, with GI values ranging from low to high (i.e., regular spaghetti ranging from 33 to 98; wholegrain spaghetti ranging from 53 to 101) [18,24]. Results from an interlaboratory study from seven research centers (Toronto, Canada; Lund, Sweden; Sydney, Australia; Milan, Italy; Dunedin, New Zealand; Trinidad, West Indies, and Potchefstroom, South Africa) found spaghetti to be a low GI food (mean value obtained from 6 centers = 47), with ranging values of 36–70 [25]. Factors influencing the GI of spaghetti include the flour used, the thickness, the cooking time, other ingredients, such as proteins, soluble fiber or fat, production processes [2,25,26], all of which may lead to differences in the size of starch gelatinization and GI values. Spaghetti products, particularly novel ones with non-cereal ingredients have a specific importance for Mediterranean agri-food production, as they may be richer in nutrients such as protein and fibers.

The aims of this study were to investigate the short-term effects of three types of spaghetti No 7 (regular spaghetti, wholegrain spaghetti, and spaghetti high in soluble fiber and low in carbohydrates) on postprandial glycemic responses.

## 2. Materials and Methods

### 2.1. Subjects

Fourteen healthy subjects (4 men, 10 women), between 18–55 years, were recruited by a variety of methods, including online advertisements and flyers and notices posted around the university campus. Subjects underwent an initial screening and measurements included anthropometry (height, weight, waist, and hip circumference), fat percentage via bioimpedance analysis (InBody 230), blood pressure (Omron, Intellisense, HEM-907, Omron Hellas, Athens, Greece), and fasting blood glucose via finger prick (MediSmart^®^ Ruby glucose meter with a lancing device, Lilly-PHARMASERV SA, Athens, Greece). Additionally, a questionnaire on general health was completed. Subjects were non-smokers, had a healthy body mass index (BMI), a normal blood pressure, and no medical conditions (i.e., cardiovascular diseases, diabetes mellitus, polycystic ovary syndrome, liver diseases, nephropathy, clinical depression, gastrointestinal disorders), were not pregnant/lactating, or taking medications known to affect glycemia (glucocorticoids, metformin, thyroid hormones, thiazide diuretics), and were not allergic to the test foods. All fourteen subjects completed all treatments and were included for analysis.

The study was conducted at the Laboratory of Dietetics and Quality of Life, Agricultural University of Athens, Greece. All subjects gave their informed consent for inclusion before participating in the study. The study was conducted in accordance with the Declaration of Helsinki, and the protocol was approved by the Bioethics Committee of the Agricultural University of Athens (EIDE Reference Number: 2021/49). This trial was registered at Clinicaltrials.gov (NCT05197283).

### 2.2. Study Design

**Figure 1:**
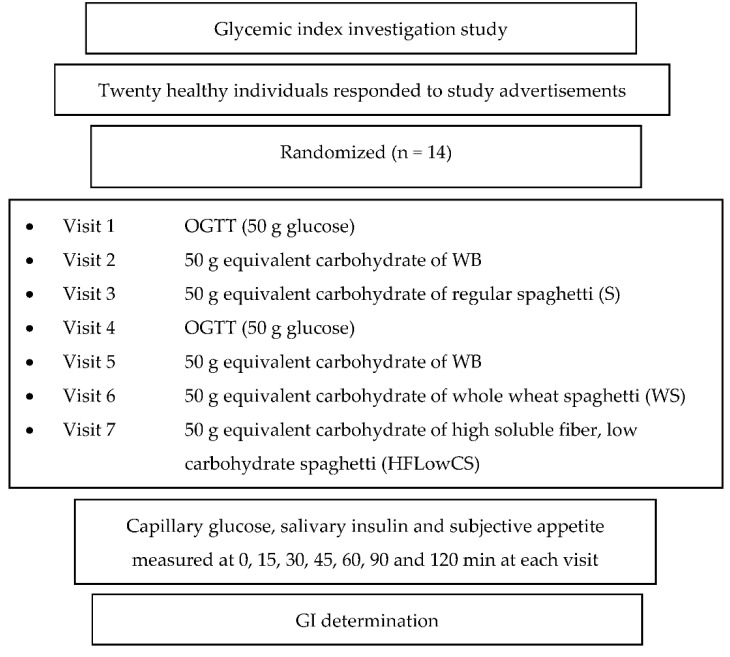
Glycemic index (GI) determination study.

The glycemic indexes (GIs) of three commercial Spaghetti No 7 pasta samples (Melissa Kikizas S.A., Athens, Greece, brand called Melissa^®^) were evaluated. The GI was determined according to ISO 26642:2010 [1], Brouns et al., (2005) [4], and the FAO/WHO (1998) [2] method and procedures. The study consisted of seven dietary treatments in a randomized, cross-over design: two glucose reference drinks, two white bread (WB) reference foods, a semolina spaghetti No 7 (S; Athens, Greece, Melissa^®^), a wholegrain spaghetti No 7 (WS; Athens, Greece, Melissa^®^), and a semolina spaghetti No 7 high in soluble fiber and low in carbohydrates (HFlowCS; Athens, Greece, Melissa^®^). Subjects attended seven test sessions of around 3 h, separated by a wash-out period of at least two days. Each test session consisted of a test meal that had to be consumed within 15 min and 2 h post-consumption measurement of metabolic blood parameters. Subjects arrived at the test center around 08:45–9:00 h in the morning following an overnight fast of 10–14 h. In addition, subjects were instructed to refrain from strenuous physical activity and alcohol on the day before the test and were only allowed to eat the provided foods throughout the test sessions. Online computer software (Social Psychology Network, Middletown, CT, USA) was used for simple randomization of the sequence of the test foods (http://www.randomizer.org/ (accessed on 1 April 2020) [27]. A researcher not involved in the collection and analysis of the scientific data, was responsible for the randomization of the volunteers to the intervention days examining the test foods. Subjects received, in a random order, the reference food (D-glucose), tested twice (i.e., 1st 4th visit) and white bread (WB), tested twice (i.e., 2nd and 5th visit) (Figure 1), and the three spaghetti products: S, WS, and HFLowCS, tested once, in different weeks, with a random sequence in accordance with the recommended GI methodology [1,2].

### 2.3. Test Meals

During each of the seven test sessions, subjects consumed one of the following test meals: S made with durum hard wheat semolina flour (Melissa^®^, Athens, Greece), WS made with wholegrain hard wheat flour (Melissa^®^, Athens, Greece), HFlowCS made with durum hard wheat semolina flour, rice bran, oat fibers, and flaxseed flour (Melissa^®^, Athens, Greece), or glucose reference drink (50 g anhydrous glucose dissolved in 250 mL water), or white bread as a second reference food. All the test foods and the reference foods were given in portions containing 50 g available carbohydrates. Regarding the pasta samples, the available carbohydrates were determined at the final cooked product (ready to eat boiled pasta), since it is known that the composition of pasta is significantly altered during boiling due to cooking losses [28]. Unsalted boiling water was chosen to cook the pasta to the recommended by the producer cooking times (i.e., recommended 8–9 min for S, selected: 8.5 min; recommended 9–10 min for WS, selected: 9.5 min; and recommended 8–9 min for HFlowCS, selected: 8.5 min cooking time). The total meal characteristics and macronutrients composition of the dried test foods based on their food label is shown in Table 1. The nutritional characteristics of the studied pasta products were evaluated in terms of their total protein content (Kjeldahl AACC 47–12), ash content (AOAC 923.03), moisture (AOAC 930.15), available carbohydrates, and total dietary fibers (Megazyme kit-K-ACHDF, Megazyme Ltd., Scotland, UK) (Table 2), as a function of their cooking time (based on the recommended cooking times of the producer). The portions of spaghetti were quickly served without adding cheese or any sauce. Portion sizes served for the test meal were based on the consumption of 50 g of products’ available carbohydrates after boiling (163.67 g portion size for boiled S; 186.26 g portion size for boiled WS; 223.06 g portion size for boiled HFlowCS) and for WB (91.40 g). The available carbohydrates were determined with the aforementioned Megazyme kit, which calculates only the carbohydrates that can be absorbed (sugars and digestible starch), neglecting dietary fiber and resistant starch.

Subjects rated their hunger, desire to eat, and perceived fullness after eating on 100 mm line visual analogue scales (VAS), ranging from not at all (0 mm) to extremely (100 mm), with for example neither hungry (0 mm), full (100 mm), or having desire for food in the middle (50 mm). VAS were given in the form of a booklet, one scale per page [29]. VAS ratings were obtained at times 0, 15, 30, 45, 60, 90, and 120 min post-test meal consumption.

### 2.4. Blood Glucose and Salivary Insulin Concentrations

After a fasting blood sample, subjects ate a test meal at a comfortable pace within 15 min and had further blood samples at 15, 30, 45, 60, 90, and 120 min after starting to eat. Participants were instructed to consume the glucose drink at a comfortable pace within 10 min. Test meals were served with 300 mL water as a drink in all seven trials.

To determine blood glucose concentrations, trained individuals from our research team, performed the capillary blood glucose monitoring procedure by skin pricking according to the scheduled time. To standardize all data collection procedures, capillary blood glucose monitoring was performed at the fingertip (distal phalange of the third finger). Capillary blood samples were collected at baseline (time 0) and at 15, 30, 45, 60, 90, and 120 min after test food or white bread or D-glucose consumption. Blood glucose was measured with glucose dehydrogenase–FAD test strips (Ruby Blood glucose Test Strips, Lilly-PHARMASERV SA, Athens, Greece), which show no reactivity to any sugars other than glucose and have better heat resistance and oxygen resistance. The allowed deviation limits of glucose meters for glucose results ≥ 100 mg/dL were within 15% of the reference method. The coefficient of variation (CV, %) was less than 5% both in intermediate precision and repeatability. The blood glucose value recorded was the mean of three measurements.

The measured glycemic values were used to build the curve of the glycemic response for every volunteer and for every tested food, including the reference foods. Then, for each sample and each study subject, iAUC was calculated geometrically, using the trapezoid rule, and ignoring the area beneath the baseline [1,2]. The GI calculation for each pasta sample used the method referred to as the mean of the ratios. For each subject, the ratio between the individual iAUC after consuming the pasta sample and the iAUC for the same subject after consuming the reference foods was calculated and expressed as a percentage value. Then, the GI of each spaghetti type was calculated as the average value of the ratios across all the subjects consuming the pasta sample [1,2]. The mean, s.d., and coefficient of variation (CV = 100 X s.d./mean) of the AUC of each subject’s repeated glucose (reference food) were calculated. The GL (g glucose equivalents)/1000 kJ values were calculated by multiplying the amount of carbohydrate contained in a 1000 kJ portion of that food (53.09 g available carbohydrate in boiled S; 47.41 g available carbohydrate in boiled WS and 37.20 g available carbohydrate in boiled HFLowCS), which was then divided by 100.

To determine salivary insulin concentrations, salivary samples using the Salivette method (Sarstedt AG and Co., Numbrecht, Germany) were taken at baseline and at 15, 30, 45, 60, 90 and 120 min after meal consumption. Before the collection of samples, volunteers washed their mouths with clear water to avoid food contamination. Then, they were asked to remove the cotton from the tube and press it with their tongue for approximately 1 min to collect saliva from all glands. The tubes were centrifuged (3000× *g* for 5 min) and stored at −80 °C. Salivary insulin concentrations were determined using the Human Insulin ELISA Kit (ALPCO, 80-INSHU-E10.1, Salem, NH, USA) based upon a sandwich-type enzyme-linked immunosorbent method. Plasma insulin and salivary insulin have been found to be significantly correlated (r = 0.882, *p* < 0.001) [30].

### 2.5. Blood Pressure Measurements and Dietary Intake Analysis

Blood pressure (BP; systolic and diastolic) was measured at the beginning and end of each intervention using an upper arm digital BP monitor (Omron, HEM-907, Omron Hellas, Athens, Greece). Participants were rested for 5 min in the supine position after which three BP measurements were taken at 1 min intervals, with the three readings averaged.

Dietary intake was assessed by 24 h recalls at every visit, and analyzed using the Diet Analysis Plus program, as well as using Hellenic and European Food Composition Databases (http://www.eurofir.org/foodinformation/food-composition-databases-2/ (Accessed on 1 April 2020). The databases were modified to include new foods and recipes.

### 2.6. Statistical Analysis

Data distribution was tested using kernel density plots. Normally distributed continuous variables are presented as the mean values ± standard error of the mean (SEM), unless otherwise stated, and the skewed as median (first tertile, third tertile). Differences in baseline continuous variables were evaluated using analysis of variance (ANOVA) for normally distributed continuous variables, Kruskal–Wallis test for skewed continuous data, and Pearson chi-square test for categorical variables. According to the ISO method 26642:2010 for GI calculation [1,2], we tested for outlying GI values in order to be excluded from the analysis. Between treatments, ANOVA for a 2 × 2 crossover study was conducted for blood glucose and salivary insulin. In a 2 × 2 design, we assume that there are no group effects since a complete randomization process was followed for treatment allocation. The models included the factors “subject” (id), “sequence” for inter-subject variation, and “period” and “treatments” to account for intra-subject variability. Time × test meal interaction was evaluated. Multiple comparisons between the interventions were tested post hoc using the Tukey test with Bonferroni correction. For all other parameters, one-way ANOVA was used to investigate differences between test meals followed by post hoc Tukey test and Bonferroni correction. Differences in VAS ratings were evaluated using one-way ANOVA and Friedman’s test. Correlations between GI and the characteristics of the test meals were determined with Spearman’s rho coefficient. The intervention trials were designed to have 80% power to detect a clinical difference of 25% for 0–120 min iAUC for blood glucose between the test and reference meals (α = 0.05). A total of ten volunteers were required for the reference and test meals to achieve the power and clinical difference. In our study, 14 participants were recruited, and each participant served as a control for themselves. Statistical significance was determined to be *p* < 0.05. All analyses were performed using SPSS software (version 23.0, SPSS Inc., Chicago, IL, USA).

## 3. Results

### 3.1. Subjects’ Characteristics

The subjects’ characteristics can be found in Table 3. There were no intermittent missing values or dropouts.

### 3.2. Glycemic Index (GI) of Three Spaghetti Types

The results of GI and GL for the three spaghetti test meals are presented in Table 4. The results revealed, similar GI values for the three tested samples (Table 4). According to the current classification [1,2], all three pastas should be considered low GI starchy foods. Based on the results, cooking led to a slight but statistically significant decrease in total proteins and minerals (ash content). In contrast, the bioavailable carbohydrates and the total dietary fiber content did not present significant differences compared to the raw product (*p* > 0.05). After statistical analysis, two individual GI values out of 98 (0.02%) overcoming the mean by at least 2 SD were excluded from the mean calculation, as specified in the ISO methodology [1,2]. GL values were calculated for 1000 kJ and not related to portion, as previously suggested [18] because it is difficult to define the serving size for each item since portion sizes vary markedly among food industries and consumers. Expression of the effect of foods on postprandial glycemia on an isoenergetic basis is a logical and practical approach [18]. Compared to glucose and WB, all three spaghetti types, S, WS, and HFLowCS, had significantly lower GI and GL values, without significant differences between them (*p* > 0.05; Table 4).

### 3.3. Blood Glucose and Salivary Insulin Concentrations

**Figure 2:**
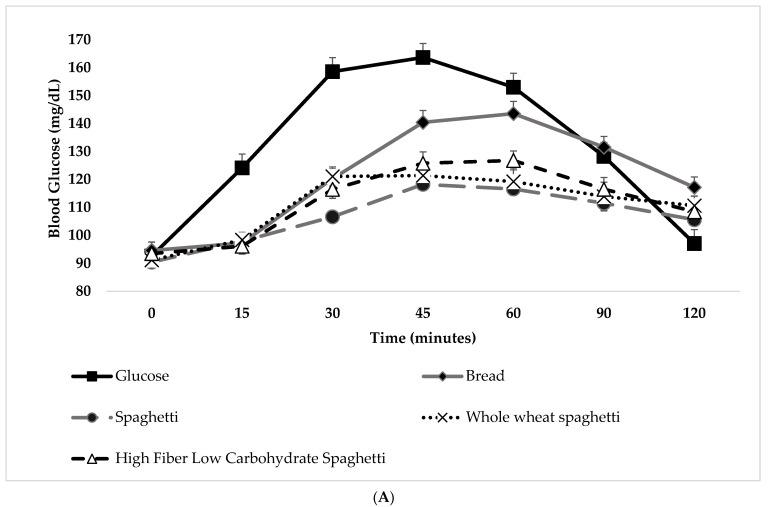

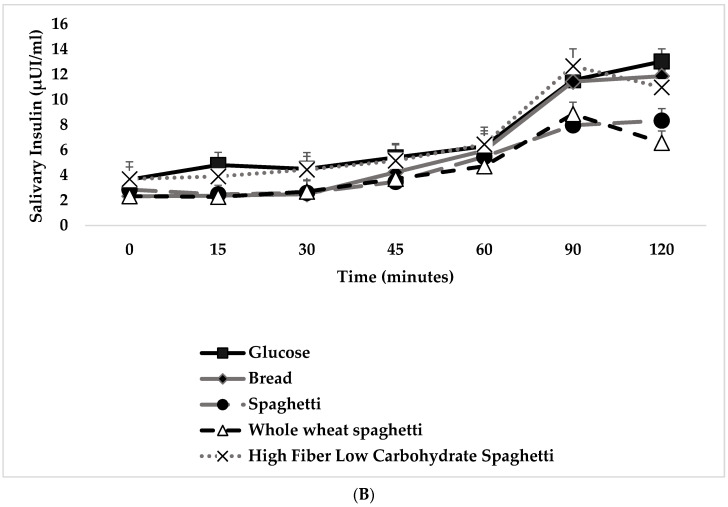
(**A**) Glycemic response after consumption of the reference food (D-glucose) and bread (white bread) and the three spaghetti food products (*n* = 14). Data are the means ± S.E.M. Post hoc Tukey test, with Bonferroni correction, was conducted to determine test meal differences at each specific time. (**B**) Insulinemic response after consumption of the reference food (D-glucose) and bread (white bread) and the three spaghetti food products (*n* = 14). Data are the means ± S.E.M. Post hoc Tukey test, with Bonferroni correction, was conducted to determine test meal differences at each specific time.

The change in postprandial glucose and insulin over time (120 min) can be seen in Figure 2A,B. No significant differences were observed in fasting glucose concentrations between glucose and WB and the test meals (*p* for all >0.05; Figure 2A). There was a significant blood glucose x time x test meal interaction (F(36,690) = 4.704, *p* < 0.001). There was a significant main effect of test meal on blood glucose concentrations (F(6,115) = 12.387, *p* < 0.001). Compared to the reference food (D-glucose), lower blood glucose concentrations were observed after the consumption of WB at 15 min, 30 min, 45 min, and 60 min (*p* for all <0.001). It is clear that all three spaghetti test meals resulted in lower glucose, but not insulin, responses over time compared to the reference foods. In particular, the glucose concentrations were lower at 15, 30, 45, and 60 min after the start of the meal for all three spaghetti test meals as compared to the reference food (D-glucose) (*p* for all <0.001), without differences between them (Figure 2A). Compared to the reference food (D-glucose), lower blood glucose concentrations were observed after the consumption of S and WS at 90′ (*p* for all <0.001; Figure 2A), and higher for WS at 120′ (*p* = 0.021; Figure 2A). Compared to WB, lower blood glucose concentrations were observed after the consumption only of S at 30′ (*p* = 0.009; Figure 2A) and 120′ (*p* = 0.043; Figure 2A). Compared to WB, lower blood glucose concentrations were observed after the consumption of S, WS, and HFLowCS at 45′ (*p* = 0.001, *p* = 0.004 and *p* = 0.027, respectively; Figure 2A), 60′ (*p* < 0.001, *p* = 0.001 and *p* = 0.017, respectively; Figure 2A) and 90′ (*p* = 0.004, *p* = 0.010 and *p* = 0.028, respectively; Figure 2A). Peak glucose values were significantly lower for all three spaghetti food products compared to the reference food (D-glucose) or to the WB (*p* for all <0.001) (Table 4). S produced a significantly lower peak glucose value compared to WS and HFlowCS, without significant differences between the WS and HFlowCS (Table 4). The 0–120 min iAUC for blood glucose values calculated for each test meal are shown in Table 4. There was a significant main effect of test meal on 0–120 min iAUC for blood glucose (F(6,117) = 8.886, *p* < 0.001). The mean within-individual variation of 0–120 min iAUC for blood glucose for the repeated tests was 36%. The 0–120 min iAUC for blood glucose values calculated for WB, S, WS, and HFLowCS were significantly lower than those of the reference food (D-glucose) (*p* for all <0.05), without significant differences between them (Table 4). The 0–120 min iAUC for blood glucose values calculated only for S were significantly lower than those of the reference food (WB) (*p* = 0.036; Table 4). No significant differences were observed for salivary insulin concentrations between meals, compared to the reference food (D-glucose) and WB at all time points (*p* for all >0.05; Figure 2B). No differences were observed for 0–120 min iAUC for salivary insulin, peak salivary insulin value, and time to peak salivary insulin value (Table 4, Figure 2B).

### 3.4. Blood Pressure and Subjective Appetite

No differences were observed for subjective appetite assessment variables or BP measurements (systolic and diastolic) between meals compared to the reference food (D-glucose) and WB at all time points (*p* for all >0.05) (data not shown).

## 4. Discussion

By applying the standard GI methodology, this study produced data for equally thin spaghetti pastas (No 7) differing significantly in dietary soluble fiber and protein content. The results showed that the glycemic responses of the three tested spaghetti pastas were significantly lower than the responses of a glucose drink or white bread as reference foods, without significant differences between them.

### 4.1. Glycemic Index (GI) and Glycemic Responses: Fiber and Protein Implications

In general, the consumption of spaghetti pasta, particularly wholegrain spaghetti or novel spaghetti pastas containing high amounts of soluble fiber and plant protein, is linked to ameliorated glycemic response and improved insulin sensitivity, partly attributed to fiber [31]. Short-term intervention studies [32] and epidemiological and prospective studies have consistently shown an association between wholegrain consumption and a reduced mortality and metabolic disease risk [33,34]. A short-term study using continuous glucose monitoring showed that young healthy adults following a low GI diet had lower average day-long glycemia compared with a macronutrient-matched high GI diet, indicating the use of low GI diets to reduce the risk of developing glucose intolerance [35]. The use of GI for the classification of carbohydrate-rich foods has been endorsed by the FAO/WHO, who recommended that the GI of foods should be considered together with information about food composition to guide food choices [2]. Consumption of foods and meals that induce a lower glycemic response and delay gastric emptying, thus leading to decreased insulin requirements and postprandial glucose excursions, has been proposed as an important strategy to ameliorate postprandial hyperglycemia and insulin resistance [36]. Such foods typically contain high fiber, particularly soluble fiber, low amounts of easily absorbable carbohydrates, and are high in proteins [36,37,38]. It has been shown that consumption of fibers (i.e., beta-glucans, whole-grain cereals) may slow the rates of gastric emptying and intestinal glucose absorption, thus reducing postprandial glucose responses in a dose-dependent manner [39]. Lowering the GI of a food may be significant as it has been shown that consumption of low GI foods may be sufficient to achieve a lower glycemic response from one meal to the next [40,41,42,43]. In 2017, the American Diabetes Association proposed consuming daily wholegrain cereal products along with weight loss for the prevention of type 2 diabetes [44].

The GI of spaghetti pastas may be affected by many factors, including the addition of soluble fiber, resistant starch, fats, proteins, processing, preparation and cooking methods, the physical form of the food, the type of sugars and starch, the ripeness or the maturity of the raw ingredients, etc. [6,18,23,24,45,46]. It has been shown that different brands of the same type of pasta, i.e., shape or size of circumference, may look and taste almost the same, but differences in the type of flour used, the technological aspects (time/temperature/humidity drying cycles; extrusion dies) and the cooking time can result in differences in the degree of starch gelatinization and consequently the GI values [18,26]. This was evident comparing the GIs of three analyzed spaghetti (i.e., regular, GI = 51; No 5, GI = 33; and No 12, GI = 50), all producing lower GIs [18]. The current evaluation showed that all three types of spaghetti pastas (semolina spaghetti, wholegrain spaghetti, and semolina spaghetti high in soluble fiber and low in carbohydrates) can be classified as equally low GI food products with a GI of 33, 38, and 41, respectively. All three had significantly lower incremental glucose responses, glucose excursions, and GIs as compared to the reference foods. Our results are in agreement with others reporting that spaghetti produces a significantly smaller rise in blood glucose compared to breads (whole grain or white) or other types of pasta, indicating that differences in food form, independent of fiber content, may have marked effects on postprandial glycemia [18,24,26,45,47,48,49]. Our results also agree with the International Food Tables [50,51]. One study examining the effects of semolina spaghetti, wholegrain spaghetti, wheat-based mee pok noodles, and rice, showed that both spaghetti pastas led to significantly lower glycemic and insulinemic responses compared to noodles and rice, without differences between the spaghetti pastas, although the wholegrain spaghetti studied contained more than double the amount of fiber compared to regular semolina spaghetti pasta [49]. Notably, in the current evaluation, there was no significant difference in glycemic responses between the three spaghetti pastas, although WS and HFlowCS contained almost four times and twelve times more, respectively, the amount of fiber. All three spaghetti pastas also produced a lower GL value compared to the reference foods without significant differences between them. Thus, our data do not support the popular idea of a reduced glycemic response elicited by increasing fiber content in foods, such as in the case of spaghetti pastas, which is in agreement with others [26,46,47,49,52,53]. The inconsistencies reported for fibers’ effects on postprandial glycemia may be due to the fact that when soluble fibers are added to foods, their molecular weight vary and interactions with other compounds can occur. It may also be that only certain types of fiber, mainly soluble (i.e., vegetable gums, derived from fruits, legumes, and psyllium), may influence the GI of foods through a reduced rate of gastric emptying as they make the chyme (partly digested food coming from the stomach) more viscous [46,53]. Other possible explanations may be that glycemic response is not significantly related to soluble dietary fiber content or that soluble fibers’ effects are masked when they interact with other compounds, or that they are not as important in controlling postprandial glycemia. Finally, it may also be considered that the postprandial glucose-lowering effects of fiber, particularly soluble fiber, will be markedly observed when added to some high GI foods, such as breads [46,53,54], and not to low GI foods, such as spaghetti pastas.

There is evidence that the acute glycemic response to whole-grain foods is greater when grains have been finely milled [55]. One study showed that the glycemic response to four different whole-grain wheat breads was related to the degree of grain processing in adults with type 2 diabetes [56]. Thus, the lack of differences observed between and among the three spaghetti pastas investigated in the current evaluation may be due to the use of hard wheat flours in all of them [26]. It has been suggested that the protein–starch matrix itself, owing to the production process, is the main basis for the reduced glycemic responses of pasta and this warrants further research [49]. It may also be that the food structure of spaghetti is the main driver of its favorable metabolic properties [49].

Another reason for the beneficial effects of spaghetti products on postprandial glycemia and GI may be due to their larger particle size which may be related to the altered starch surface area available for enzymatic action [57,58]. Our group [23] and others [59,60] have shown that bran larger particle size leads to lower GI, GL, and glycemic responses compared to bran smaller particle size. Gluten partially protects starch from digestion [6]. Thus, the increased particle size may lead to decreased starch susceptibility to hydrolysis, lower starch digestion, and delayed gastric emptying [59,60,61]. It has been shown that the rate of gastric emptying is not influenced by soluble fibers, but by other factors, such as bran particle size and the meal’s energy density [62]. A study investigating the rate of gastric emptying and the glycemic response after consumption of wheat and oat-based breakfast meals showed that wheat meal caused delayed gastric emptying and improved glycemic response compared to oatmeal, despite the presence of β-glucan in oats [62]. Moreover, the beneficial results of spaghetti products on postprandial glycemia may be due to their lower hydration status, since it has been shown that foods with lower hydration (i.e., 70% of water content) produced the lowest estimated GI and GL values [17]. We have previously reported a negative correlation of dough water content with GI [23].

Meal composition and ingestion of low GI foods, particularly when consumed along with protein and/or fat, reduce postprandial glucose and insulin responses, thus improving insulin sensitivity [37,38,63,64,65,66], which may be due to their insulinotropic effects. It has been reported that 50 g protein addition to a carbohydrate food, such as white bread, can significantly lower the GI of the meal by 27% and the incremental area under the curve for glucose by 27% [66]. However, another study in patients with type 1 diabetes examining the effects of two types of pasta, a higher protein pasta containing 10 g protein/serving and regular pasta with 7 g protein/serving compared to extra-long grain white rice (all meals containing 42 g carbohydrates) on glucose responses using continuous glucose monitoring, showed that both pasta compared to white rice led to significantly lower peak glucose levels and lower total glucose AUC, without significant differences between them [48]. Other studies suggested that pasta formulated with legume flours, red lentils, grass rea and chickpea, produced lower GIs (GI ranging from 20 to 58) compared to wheat pasta (GI ranging from 53 to 73), possibly due to the two to three times higher levels in fiber and proteins (24–27 g/100 g) with respect to wheat pasta (14 g protein/100 g) [67,68]. Two studies from our group, an acute [69] and a short-term (8 weeks duration) [70] cross-over randomized clinical trial, and a 2013 meta-analysis of studies ranging from 4–24 weeks, reported that high-protein eating plans (25–32% of total energy vs. 15–20%) resulted in two kilograms greater weight loss, better maintenance of muscle mass and 0.5% greater improvements in HbA1c, without significant decreases in fasting glucose levels [71]. In our current evaluation, we did not observe differences in postprandial glycemic responses between the three tested spaghetti pastas, which is in agreement with others [49,72], although the tested spaghetti pastas had marked but not clinically significant differences in protein content (7 g in boiled S vs. 13 g in boiled WS vs. 10 g in boiled HFlowCS). This observation could also be because the postprandial glucose-lowering effects of protein, would be more clearly observed when added to high GI foods, such as breads, and not to low GI foods, such as spaghetti pastas.

#### 4.1.1. Blood and Salivary Insulin Responses

One study found an improved insulin response after semolina spaghetti compared to wholegrain spaghetti which confirmed that a higher fiber content in spaghetti did not affect the metabolic responses [49]. Although salivary insulin is not commonly used in clinical studies, it has been shown to be a non-invasive tool for assessing hormones’ concentrations [73,74] and has been positively correlated to serum insulin in healthy young subjects with normal body weight and overweight/obesity [30,73,74] and in people with type 1 diabetes [75]. In the current evaluation, we did not observe differences between spaghetti pastas in postprandial salivary insulin concentrations, regardless of their fiber or protein content. Our results are in agreement with two studies showing no effects of particle size on insulin responses in healthy subjects [23,76] but are in contrast with another study showing that larger particle size produced significantly lower insulin responses in people with type 2 diabetes [77]. The conflicting results may be due to the different study design, i.e., different bread formulations, and the population under study, i.e., healthy vs. subjects with type 2 diabetes. It is reasonable that it may be easier to detect differences in a population having hyperinsulinemia and insulin resistance compared to healthy young adults participating in our study.

#### 4.1.2. Blood Pressure

Blood pressure was not found to be different at baseline and end of all trials between and among the test meals studied. It is well known that foods’ salt content and addition of salt to foods have a significant impact on BP. In our study, all spaghetti test meals contained low amounts of sodium and were prepared in the same manner with unsalted water, which may explain the lack of differences between and among meals regarding BP. Moreover, our subjects were young and normotensive, which may also be significant factors for not observing acute differences in BP among and between test meals. Similarly, we did not observe acute differences in subjective appetite between and among test meals, regardless of their protein or fiber content, which may be due to the limited time frame of two hours duration of our study.

### 4.2. Study Limitations and Advantages

Among the limitations of the study, blood collection from the participants enabled measurements of plasma insulin and incretins. The strength of our study was the randomized crossover design where each subject served as his own control.

### 4.3. Practical Applications

To the best of our knowledge, this study determined for the first time the GI of three different types of spaghetti pastas, differing significantly in soluble fiber and protein. Our study followed controlled circumstances for a GI protocol. Based on our results, spaghetti pastas, particularly whole grain, and novel ones with low total carbohydrates, high in soluble fiber and protein are advisable for the prevention of high blood glucose excursions and should be considered by people with overweight/obesity or type 2 diabetes. It should be stressed out that the novel type of spaghetti contains twelve times more dietary fibers compared to the simple spaghetti and triple the dietary fibers compared to the whole grain one. The high content of dietary fibers attributes to the overall daily intake of fibers, with a portion of 100 g covering almost 80% of the recommended daily intake of dietary fiber according to the WHO. Moreover, the HFLowCS can be a useful food item for patients with type 2 diabetes as in the same portion size with simple and whole-grain spaghetti, they take 65% and 30% less carbohydrates, resulting in a better glycemic response and lower need for insulin secretion. Moreover, the higher plant-based protein content alongside the high soluble dietary fiber makes the HFLowCS a novel food item with significant importance for the management of cardiometabolic risk and the overall health of the gut.

## 5. Conclusions

In conclusion, the results of this study confirmed that spaghetti pastas, regardless of their soluble fiber and/or protein content are low GI foods, producing lower postprandial glucose concentrations and lower glucose excursions in young healthy subjects, indicating that they are a suitable dietary alternative for glycemic control. Future long-term studies are needed to provide an insight regarding the mechanisms by which different types of pasta of varied formulations elicit a more favorable impact on the glycemic response in different population groups including subjects with type 2 diabetes and/or obesity. The efforts of the food industry to improve the nutrient density of foods and their impact on disease prevention, such as spaghetti containing twelve times more dietary fibers compared to regular spaghetti and three times more fiber compared to whole grain ones, are highly encouraged as they can aid significantly in covering almost 80% of the recommended daily intake of dietary fiber according to the WHO, whilst consuming 65% less carbohydrates than regular spaghetti and 30% less carbohydrates than whole-grain spaghetti in the same portion consumed. Consumption of spaghetti pasta, particularly whole-grain ones and novel ones with different formulations, high in soluble fibers and plant proteins, is an important strategy for glycemic control and prevention or dietary treatment of chronic diseases.

## Figures and Tables

**Table 1:** Macronutrient composition per 100 g described based on the food label.

	Semolina Spaghetti No 7	Wholegrain Spaghetti No 7	High soluble Fiber Low Carbohydrate Spaghetti No 7
Energy (kcal)	1500 kJ/354 kcal	1502 kJ/355 kcal	1398 kJ/333 kcal
Fat (gr) Saturated fat (gr)	1.5 0.3	2.1 0.4	4.6 0.8
Carbohydrates (gr) Sugar (gr) Polyols (gr)	72 3.8 11	67.6 2.6 12.8	47.4 2.2 5.4
Dietary fiber (gr)	1.8	7.0	21.1
Protein (gr)	12	12.8	14.9
Salt (gr)	0.03	0.15	0.18

**Table 2:** Nutritional analysis of test spaghetti meals.

Spaghetti Type		Cooking Time * (min)	Protein Content (%DM)	**Ash Content** **(%DM)**	**Available Carbohydrates** **(%DM)**	**Total Dietary Fibers** **(%DM)**
Spaghetti	Raw		10.16 ± 0.15 ^a^**	0.93 ± 0.04 ^a^	84.99 ± 1.65 ^a^	1.82 ± 0.08 ^a^
	Cooked	8.0	7.20 ± 0.11 ^b^	0.66 ± 0.02 ^b^	84.04 ± 1.29 ^a^	1.79 ± 0.06 ^a^
		8.5	6.98 ± 0.13 ^c^	0.62 ± 0.02 ^b,c^	83.01 ± 1.56 ^a^	1.77 ± 0.07 ^a^
		9.0	6.63 ± 0.12 ^d^	0.59 ± 0.03 ^c^	81.74 ± 1.83 ^a^	1.79 ± 0.04 ^a^
Wholegrain Spaghetti	Raw		14.97 ± 0.13 ^a^	1.65 ± 0.05 ^a^	75.94 ± 1.15 ^a^	7.04 ± 0.06 ^a^
	Cooked	9.0	13.43 ± 0.10 ^b^	1.24 ± 0.01 ^b^	75.43 ± 1.53 ^a^	7.01 ± 0.05 ^a^
		9.5	13.26 ± 0.07 ^b^	1.22 ± 0.01 ^b,c^	74.99 ± 1.66 ^a^	6.96 ± 0.07 ^a^
		10.0	12.79 ± 0.08 ^c^	1.21 ± 0.01 ^c^	73.75 ± 1.27 ^a^	6.99 ± 0.05 ^a^
High soluble Fiber low Carbohydrate Spaghetti	Raw		12.39 ± 0.09 ^a^	3.22 ± 0.03 ^a^	55.16 ± 1.62 ^a^	21.13 ± 0.09 ^a^
	Cooked	8.0	10.57 ± 0.12 ^b^	3.05 ± 0.02 ^b^	55.04 ± 1.48 ^a^	21.05 ± 0.06 ^a^
		8.5	10.04 ± 0.10 ^c^	3.03 ± 0.01 ^b,c^	54.78 ± 1.55 ^a^	21.08 ± 0.06 ^a^
		9.0	9.34 ± 0.10 ^d^	2.82 ± 0.03 ^c^	52.04 ± 1.59 ^a^	20.89 ± 0.05 ^a^

* Based on the recommended cooking times for each category. ** Values with different superscripts (a, b, c, d) were significantly different as shown by Duncan’s multiple range. test. The superscripts are referred to each one of the spaghetti types and indicate the differences among the values for each column.

**Table 3:** Baseline participants’ characteristics (*n* = 14).

Characteristics	Total
N	14 (4 men, 10 women)
Age (years)	25.21 ± 0.91
Weight (kg)	64.51 ± 4.44
Height (cm)	167.43 ± 0.10
Body mass index (BMI; kg/m^2^)	22.67 ± 0.89
Basal metabolic rate (BMR, kcal)	1534.46 ± 143.92
Body fat (kg)	14.78 ± 1.62
Muscle mass (kg)	27.16 ± 2.28
Waist circumference (cm)	77.54 ± 2.98
Hip circumference (cm)	105.89 ± 7.36
Dietary intake (from 24-h recall)	
Protein (gr)	67.0 ± 7.662
Carbohydrate (gr)	206.27 ± 21.28
Fat (gr)	65.17 ± 7.51
Saturated fat (gr)	21.58 ± 2.71
Total cholesterol(gr)	218.74 ± 26.99
Fiber (gr)	18.06 ± 1.96
Sodium (gr)	2648.74 ± 509.83
Energy intake (kcal)	1668.85 ± 165.97

Values are means ± SEM or median (first, third tertile).

**Table 4:** Incremental area under the curve (iAUC) for blood glucose, glycemic index (GI) and glycemic load (GL) of three spaghetti food products, relative to the reference D-glucose and white bread (WB).

Food (Serving Size Containing 50 g Available Carbohydrates)	iAUC (mmol 120 min l^−1^)	GI (Glucose as Reference Food)	GI (WB as Reference Food)	GL (Glucose as Reference Food)	GL (WB as Reference Food)	Glucose Peak Value (mg/dL)
Glucose	4478 ± 228 ^a^	100 ^a^	_	_	_	79.63 ± 4.23 ^a^
WB	3415 ± 228 ^b^	73.55 ± 5.47 ^b^	_	36.77 ± 2.74 ^b^	_	55.79 ± 3.53 ^b^
S (163.67 g)	2144 ± 324 ^c^	32.97 ± 4.29 ^c^	46.26 ± 5.24	17.48 ± 2.28 ^c^	24.52 ± 2.78	32.63 ± 4.23 ^c^
WS (186.26 g)	2547 ± 324 ^b,c^	38.31 ± 3.77 ^c^	48.84 ± 4.81	18.00 ± 1.77 ^c^	22.95 ± 2.26	37.44 ± 4.67 ^b^
HFLowCS (223.06 g)	2567 ± 324 ^b,c^	40.55 ± 4.37 ^c^	47.33 ± 4.35	15.00 ± 1.62 ^c^	17.51 ± 1.61	39.57 ± 3.95 ^b^

Data are the means ± SEM. Each value represents the mean of fourteen replicates. Abbreviations: WB: white bread; S: durum semolina wheat spaghetti; WS: wholegrain spaghetti; HFLowCS: high soluble fiber low carbohydrate spaghetti. Values marked with the same superscript letter are not significantly different (*p* > 0.05). Means were compared column-wise by using one-way ANOVA for factor “treatment”, period and sequence of treatment, and post hoc Tukey test with Bonferroni correction to account for multiple comparisons between test meals; *p*-values < 0.05 were considered as significant. To convert mg/dl to mmol/L, values need to be divided by the number 18.

## Data Availability

Not applicable.
